# Real-time observation of pathophysiological processes during murine experimental *Schistosoma japonicum* infection using high-resolution ultrasound imaging

**DOI:** 10.1186/s41182-017-0082-5

**Published:** 2018-01-05

**Authors:** Katsumi Maezawa, Rieko Furushima-Shimogawara, Akio Yasukawa, Nobuo Ohta, Shiro Iwanaga

**Affiliations:** 1 Department of Environmental Parasitology, Tokyo Medical and Dental University Graduate School of Medical and Dental Sciences, 1-5-45 Yushima, Bunkyo-ku, Tokyo, 113–8519 Japan; 2 Nishiogi Veterinary Medical Hospital, 4-9-2 Nishiogikita, Suginami-ku, Tokyo, 167–0042 Japan; 3 Depertment of Clinical Nutrition, Faculty of Health Science, Suzuka University of Medical Science, 1001-1, Kishioka-cyo, Suzuka-shi, Mie 510-0293 Japan

**Keywords:** *Schistosoma japonicum*, Schistosomiasis, Ultrasonography, Non-invasive observation, Liver fibrosis, Portal hypertension, Real-time imaging

## Abstract

**Background:**

Hepatosplenic lesion formation is one of the typical clinical symptoms of schistosomiasis japonica. Although it is established that circum-oval granuloma formation mediated by T lymphocytes is the key event triggering the formation of hepatic lesions, the time-course kinetics of disease progression remains to be fully elucidated.

**Methods:**

The real-time process of the pathophysiology of schistosomiasis japonica from the early to late clinical phase was non-invasively observed in a murine experimental infection model using high-resolution ultrasonography. Together with clinical parameters, including body weight and the levels of serum markers of hepatic damage or fibrosis, ultrasonography was used to assess changes in the liver parenchyma and diameter of the portal vein and portal blood flow velocity. In parallel, parasitological parameters were observed, including egg number in the feces and maturation of parasites.

**Results:**

Abnormal high-echo spot patterns in the liver parenchyma, reflecting hepatic fibrosis in ultrasonography, appeared in the liver at 4 weeks post-infection and the pattern became more enlarged and severe over time. This finding was concordant with parasite maturation and initial egg excretion. The serum M2BPGi level markedly increased from 8 weeks post-infection, suggesting sharp deterioration of hepatic fibrosis. At the same time, the diameter of the portal vein, reflecting portal hypertension, became enlarged and reached the peak level at 8 weeks post-infection. Ascites were apparent around the spleen at 9 weeks post-infection, and dilatation of the splenic vein was noted at 10 weeks post-infection. Live adult worms seemed to be detected in the portal vein at 4 weeks post-infection by ultrasonography.

**Conclusions:**

We obtained real-time imaging of the development of hepatosplenic lesions of schistosomiasis japonica in mice. The time-course kinetics of the onset, development, and modulation of each symptom was uncovered. These results are expected to provide new clues for understanding the pathophysiology of human schistosomiasis japonica.

## Background

Schistosomiasis is caused by the intravenous trematode *Schistosoma* sp., and is endemic to more than 70 countries, putting 218 million people at risk of infection. *Schistosoma japonicum* infection is endemic in Asian countries, including China, the Philippines, and Indonesia; as a zoonotic pathogen, control of *S. japonicum* infection is more complicated and difficult compared with that of other schistosome species [1, 2]. Schistosomiasis is divided into two clinical types, intestinal and urinary, and *S. japonicum* causes the former type. Schistosomiasis japonica is characterized by severe hepatosplenic lesions with circum-oval granuloma formation, hepatic fibrosis, portal hypertension, and so forth [3–5].

The immunopathology of schistosomiasis has been well characterized; several studies have shown that the T cell response to egg antigens triggers the symptoms. Parasite eggs discharged inside blood vessels are trapped in the capillary vessels, which induces granuloma formation around the vessels under the control of egg antigen-specific T cell responses [6–8]. Cercariae, the infective larvae of schistosome parasites, penetrate the host skin and mature in the mesenteric/portal vein. In a murine experimental infection model, adult *S. japonicum* start egg production around 4 weeks post-infection (PI), and thus, the hepatosplenic lesion develops as of 4 weeks PI [9]. The peak response is observed around 8 weeks PI, and then, the granulomatous response is subsequently downregulated due to initiation of an immunomodulation mechanism [10]. Discussion on the time-course development of pathology has mainly been based on cross-sectional observations because the samples were obtained from sacrificed animals at the time of observation. However, continuous changes during the infection process have not yet been studied because of the lack of available non-invasive equipment for small experimental animals. High-resolution ultrasonography is a recently available technology, which has now become applicable for studies with mice [11, 12]. As a non-invasive approach, it is possible to conduct continuous observations of disease progress in the same mouse using high-resolution ultrasonography [13, 14]. There is no direct evidence that the clinicopathological changes observed in a mouse model are comparable to those in human infection; however, the pathophysiology of schistosomiasis in humans seems to be similar to findings in the murine model. Thus, research on the pathophysiology of human schistosomiasis would be greatly promoted with the possibility to conduct longitudinal, but not cross-sectional, observations in mice.

Accordingly, the aim of the present study was to conduct real-time observations of the progress of schistosomiasis japonica using high-resolution ultrasonography in a murine model of infection. The main goal of our study was to continuously observe the time-course development of hepatosplenic lesions in a non-invasive manner in the same group of mice to obtain more detailed and dynamic information on the pathophysiology of schistosomiasis japonica. The results were compared with information obtained by conventional parasitological and/or pathological tools. Based on this new source of information, we discuss the process of disease development and modulation and the future applicability of this approach.

## Methods

### Animals

Female BALB/c mice (5 weeks old) were used in this study (Crea Japan, Tokyo). The mice were maintained at 23 °C with a 12-h light/12-h dark cycle with free access to food and water.

### Parasite infection

For short to mid-term observations up to 13 weeks PI (group 1, *n* = 12), 5-week-old mice (body weight 18–21 g) were infected with 25 cercariae of the Yamanashi strain of *S. japonicum* under intraperitoneal anesthesia with 50 mg/kg of pentobarbital as described elsewhere [15]. In parallel, another group of mice (group 2, *n* = 10) was infected with 10 cercariae for longer term observation for up to 1 year. Mice that were not infected were used as negative controls.

### Body weight and egg number per gram (EPG) counts

Body weight of the infected mice of group 1 was measured up to 13 weeks PI except for 3 weeks PI and 6 weeks PI. Worm eggs were gathered from the mice feces of each week in order, fixed the eggs with formalin, melted the residue with ethyl acetate, and then collected by MDL modification method in which the eggs were precipitated by centrifugation. Thereafter, the number of the eggs per 1 g in feces was calculated by microscopic examination (EPG). EPG was determined at 7, 8, 9, and 11 weeks PI.

### Ultrasonographic observation

Ultrasonic examination was continuously performed weekly for weeks from 3 to 13 weeks, randomly selected from group 1. For the mice randomly selected, the same individuals were used until the end of the experiment to see changes in intraperitoneal cavity and EPG over time. The mice were fixed on a platform after being anesthetized with Somnopentyl® or Isoflurane®, and ultrasonographic observation was performed while keeping the mouse in a fixed position with a conditioned temperature and stable circulation. The echo jelly warmed with hot water was applied to the same site, and the optimal position for intraperitoneal retrieval was used. For the mice in group 2, the observation was performed at 1 year PI.

We used an ALOKA Noblus (linear probe for humans, 18–4 MHz; Hitachi Corporation, Tokyo, Japan) or Prospect 3.0 ultrasound imaging apparatus for small animals (50 MHz, resolution 30 μm; Nepagene, Chiba). The ALOKA machine was used for observations over a wide range of tissues, including the liver, spleen, portal vein, intestine, and ascites. The spleen depth was measured in the middle portion of the organ. The diameter of the portal vein trunk was measured at the porta hepatis, and the diameter of the splenic vein was measured near the branch from the portal vein. The Prospect 3.0 machine was used for measuring the angle of the liver edge and portal blood flow.

### Blood markers of liver function and fibrosis

A mouse different from the mouse used in the ultrasonic examination was sacrificed by injection of a lethal dose of pentobarbital. Blood samples were obtained at 0, 6, 7, 8, 10, 13, and 23 weeks PI.

After macroscopic aspects of the livers and other abdominal organs were recorded, blood was collected from the posterior vena cava and the alanine amino transferase (ALT), albumin/globulin ratio (A/G), and Mac-2 binding protein glycosylation isomer (M2BPGi) [16–18] values were measured as indicators of liver function and hepatic fibrosis. ALT and A/G were inspected by Oriental Yeast Co., Tokyo and M2BPGi was inspected with Possible Co., Gifu.

### Statistical analysis

Statistical analysis was performed with Student’s *t* test. *P* values < 0.05 were regarded as statistically significant.

## Results

### Body weight and EPG

**Fig. 1:**
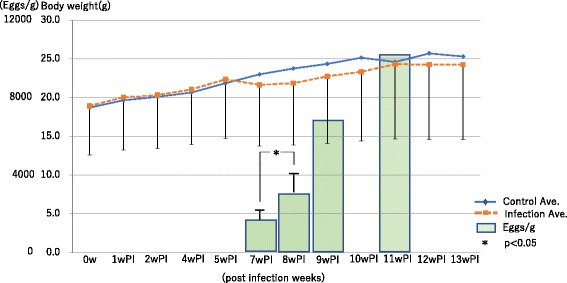
Observation of body weight and EPG. Dotted line lines indicate changes in body weight of infected mice and solid line lines indicate control mice. The bars showed EPG. Differences in body weight seemed to be apparent between infected mice and control mice after 7 weeks PI. However, there was no significant difference between control and infected mice

There was no difference in body weight up to 6 weeks PI, but differences in body weight became apparent between infected mice and control mice as of 7 weeks PI (Fig. 1). Although the difference was only statistically marginal, this trend suggests the progression of general symptoms as of 6 weeks PI. An increase of EPG was also observed on the infected mice at 7 weeks PI, and EPG continuously increased over time (Fig. 1).

### Blood parameters

**Fig. 2:**
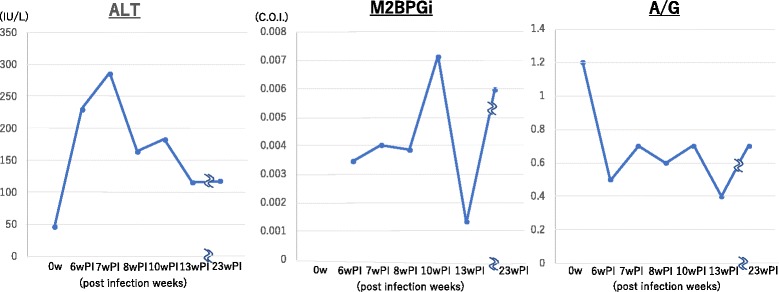
Liver function and fibrosis. The peak of ALT (IU/L) was 7 weeks PI, and the start of the rise of M2BPGi (C.O.I.) was observed at 8 weeks PI. The A/G ratio was markedly reduced at 6 weeks PI and was stable thereafter

The ALT values increased as of 6 weeks PI, and the maximum values were noted around 7 weeks PI. The M2BPGi level, as a marker of hepatic fibrosis, was increased from 8 weeks PI. After that, it decreased markedly at 13 weeks, again high at 23 weeks. The A/G ratio decreased gradually up to 6 weeks PI and was stable thereafter. Together, these results suggested that failure of liver function was most apparent around 6–8 weeks PI (Fig. 2).

### Ultrasonography

#### Liver

**Fig. 3:**
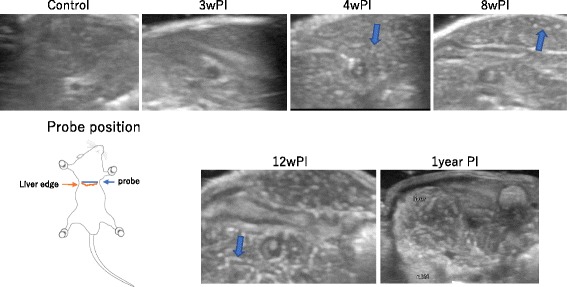
Change of the liver parenchyma in ultrasonography. At 3 weeks PI, there was no marked change in the liver parenchyma and spots appeared from 4 weeks PI (arrow). After that, the spots were enlarged, and at 8 weeks PI, a thin linear change was also detected (arrow), which became more prominent at 12 weeks PI (arrow). A thick linear change was conspicuous at 1 year PI

In the control mice, there was no particular signal in the liver, and the strength of the signal was almost at the same level between the liver and kidney through the study period. In group 1 mice, no particular change was detected before 3 weeks PI, but diffuse spots of a high-echo signal were detected at 4 weeks PI and after. These spots then increased in number, a thin linear shadow appeared at 8 weeks PI, and the spots were fused to each other and expanded in size at 10 weeks PI. These linear high-echo signal areas became more intensified and spread over the whole liver. A similar tendency was detected in the livers of group 2 mice, and the linear high-echo spots were even further intensified (Fig. 3).

**Fig. 4:**
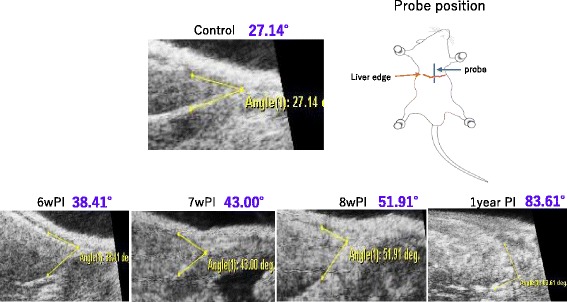
Angle of the liver edge. The angle of the periphery of the liver became gradually obtuse

Along with the progression of infection, the angle of the periphery of the liver became gradually obtuse, indicating the appearance of hepatic hypertrophy and/or atrophy (Fig. 4).

### Portal vein and splenic vein

**Fig. 5:**
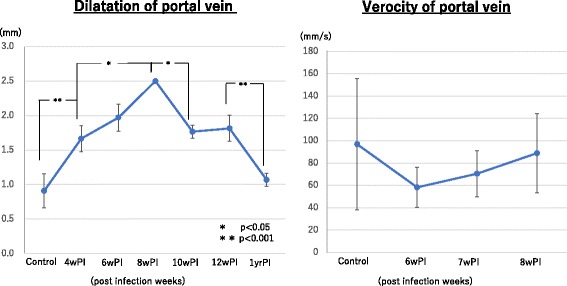
Dilatation of the portal vein and velocity. The portal vein became enlarged, which continued until 8 weeks PI and then gradually recovered to the normal range. Blood flow of the portal vein temporally slowed down at 6 weeks PI but returned back to the control level thereafter

The diameter of the portal vein of the control mice was 0.7–0.8 mm, which was enlarged in group 1 mice. Such enlargement continued until 8 weeks PI, but then gradually recovered to the normal range. The mice in group 2 (at 1 year PI) showed a portal vein diameter that was similar to that of the control mice. The blood flow of the portal vein temporally slowed down at 6 weeks PI, but returned back to the control level thereafter (Fig. 5). Of note, an unidentified image was observed inside the portal vein at 4 weeks PI and thereafter. The image was independent of the heart beat and moved slowly, suggesting that it was a live worm body.

**Fig. 6:**
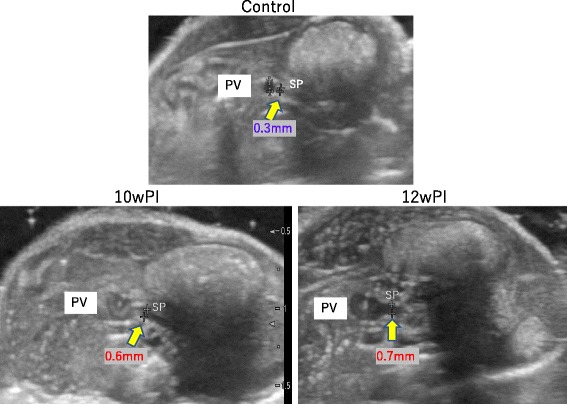
Dilatation of the splenic vein. The diameter of the splenic vein (SP) was around 0.3 mm in control mice at the site of divergence from the portal vein (PV) but doubled in size at 10 and 12 weeks PI

The diameter of the splenic vein was approximately 0.3 mm in control mice at the site of divergence from the portal vein and then doubled by 10 weeks PI (Fig. 6). A similar degree of dilatation was observed at 12 weeks PI, but it was difficult to clearly detect in the mice at 1 year PI due to the influence of intestinal flatulence.

### Other findings of the peritoneal cavity

**Fig. 7:**
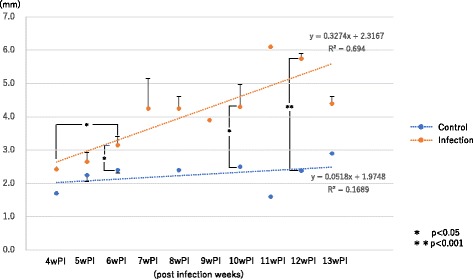
Depth of the spleen. The depth of the spleen was gradually enlarged from 4 weeks PI and then continued to increase

**Fig. 8:**
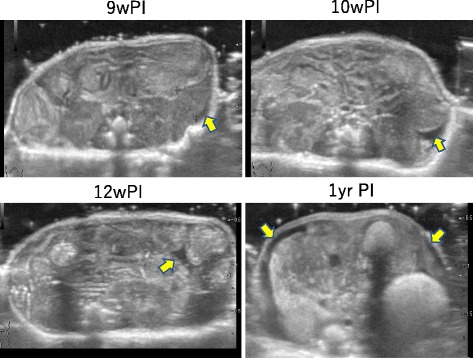
Ascites. Low-level ascites were noted at 9 weeks PI and increased gradually at 10 weeks PI and thereafter. Mice at 1 year PI showed more intense ascites. (arrow; ascites)

**Fig. 9:**
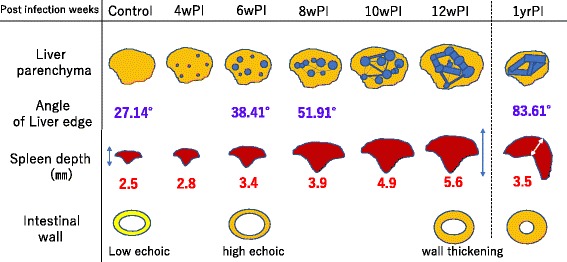
Schema of hepatosplenomegaly and intestinal lesions. Along with changes in the liver parenchyma and changes in the spleen cross-section, a high-echo signal was noted indicating intestinal wall thickening, which increased in intensity over time

The depth of the spleen became gradually enlarged from 4 weeks PI (Fig. 7). A low level of ascites was noted at 9 weeks PI, which increased gradually at 10 weeks PI and thereafter. The mice at 1 year PI showed more intense ascites (Fig. 8). Observation of the intestinal wall suggested loss of flexibility as of 6 weeks PI because a high-echo signal was noted in the intestinal wall thickening, which increased in intensity over time (Fig. 9).

## Discussion

Recent developments of tools for bioimaging have provided the opportunity to conduct real-time observations of various biological phenomena and track the responses occurring inside living bodies [19, 20]. High-resolution ultrasonography is one such tool for real-time bioimaging and has been applied in various fields of clinical medicine for diagnosis and/or clinical evaluation. High-resolution ultrasonography is already widely applied in the field of veterinary medicine, and improvement of the tools has facilitated their application in experimental mice for research.

The profiles of parasitic diseases are outcomes of complicated host–parasite interactions and require continuous/longitudinal observation rather than conventional cross-sectional observations, to provide information for gaining a more profound understanding of the pathophysiology. Although it has been elucidated that hepatic fibrosis, portal hypertension, splenomegaly, ascites, and other characteristics are typical findings of schistosomiasis japonica [21–23], the underlying pathophysiology remains somewhat of a jigsaw puzzle because each piece appears to be somewhat independent and this information has been obtained using different sample sources and approaches.

**Fig. 10:**
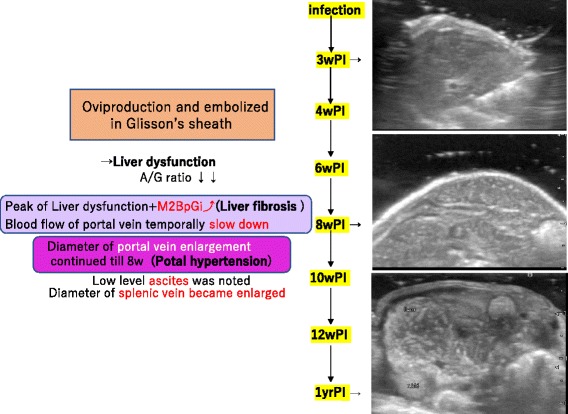
Time course of an experimental mouse model of *S. japonicum* infection*.* We were able to capture the progression of hepatic parenchyma abnormalities, secondary liver fibrosis, and portal hypertension over time with the support of blood data

In the present study, we adopted high-resolution ultrasonography to determine the time-course changes of hepatosplenic lesions formed during *S. japonicum* infection in mice, and the findings and their detailed order of each clinical sign are summarized in Fig. 10. In brief, liver dysfunction due to embolized eggs was the first response, followed by hepatic fibrosis. Hepatic fibrosis was enhanced without modulation, and thickening of the intestinal wall appeared at around the same time or slightly earlier. The thickening of the intestinal wall is more likely to be due to embolism by worm eggs [24, 25]. Portal hypertension was observed later, and the peak level was detected at around 8 weeks PI. Ascites and dilatation of the splenic vein were the final events detected; after that time point, the portal hypertension and liver function impairment returned to the control levels.

Hepatic fibrosis is one of the typical symptoms of schistosomiasis japonica. Spotty echo was detected in the liver parenchyma of infected mice at 4 weeks PI and after, which was similar to the “starry-sky”-like pattern of changes in the human liver described by the World Health Organization (WHO) [26–28]. In the WHO guidelines, the extent of liver parenchyma in ultrasonography is scored and used for evaluating the severity of schistosomiasis japonica, in the order of a “starry-sky,” “rings and pipe-stems,” “ruff” around portal bifurcation, “patches,” and “bird’s claw” pattern. In the present study, starry-sky-like lesions were observed as of 4 weeks PI, which might indicate egg granuloma formation and fibrosis of Glisson’s sheath [9]. This finding was concordant with the timing of oviproduction and egg embolism in the microcapillary vessels in Glisson’s sheath. The fibrotic pattern observed in the late phase in mice seemed to be equivalent to the “network pattern” of fibrosis observed in cases of human schistosomiasis [29–31]. Our observation of mice at 1 year PI revealed more severe fibrotic patterns, although the other parameters returned to almost normal levels.

Along with hepatic fibrosis, hypertrophy or atrophy of the liver was promoted, suggesting that the liver damage had already been promoted in the earlier phase of infection. The liver dysfunction was caused by microembolism of the eggs, resulting in a circulatory disturbance, and hepatic function was impaired from the beginning of infection. Although liver function was impaired during the phase of hepatic fibrosis, it recovered in spite of the continuous progression of hepatic fibrosis. Progress of hepatic fibrosis on ultrasound images was not a parallel trend to the transition of M2BPGi level. M2BPGI level may be similar to markers such as IL-17 and TGF-β [9].

With regard to portal hypertension, the hepatic arterial blood flow rate has been reported to increase to compensate for the decreased portal blood flow [32–34]; however, this was not apparent in our present observations. The diameter and blood flow of the portal vein suggested that the diameter and portal pressure were associated in the early phase of infection. However, hepatic fibrosis was at the maximum level around 8 weeks PI and then gradually decreased, reaching almost normal levels (despite the presence of severe hepatic fibrosis) at 1 year PI. There are various explanations for this finding. For example, portal hypertension reached the maximum level at the time point of maximum egg granuloma formation, or blood flow continued through bypass routes to eliminate the portal hypertension. As the dilatation of the splenic vein was clearly detected at 10 weeks PI, a change in hemodynamics similar to that of human portal hypertension could have occurred, which was not captured on the ultrasound during our observations. It is thus possible to speculate that portal hypertension and the degree of hepatic fibrosis are not necessarily parallel responses to infection.

It is interesting to note that our high-resolution ultrasonographic observation seemed to detect the presence of a live parasite in the portal vein. The image moved spontaneously and slowly, and the length was comparable to the size of adult *S. japonicum*. If this image was indeed an adult worm, and if it would be possible to obtain more clear images, high-resolution ultrasonography can emerge as a new diagnostic approach. From the perspective of parasitology, it is still not clear how the schistosomulae move from the host lung to the mesenteric/portal vein. Thus, real-time bioimaging might be a useful tool for resolving this long-standing question.

The real-time bioimaging of the pathophysiology of schistosomiasis japonica can also provide a new tool for research on vaccine/new drug development. Currently, the efficacies of a vaccine and/or new drug with respect to reduction in the worm burden or alleviation of the pathological damage are analyzed with conventional approaches. It is expected that new information would become available by conducting imaging analysis with ultrasonography, especially with respect to how and when the worms are killed or the pathology becomes modified in experimental murine schistosomiasis. This would further help to expand strategies of vaccine/drug development and make such control tools more feasible in the near future [35].

## Conclusions

We successfully conducted the real-time imaging of the development of hepatosplenic lesions of schistosomiasis japonica in mice. It is expected that new information will become available using imaging analysis with ultrasonography as to how and when the worms are killed or the pathology is modified in experimental murine schistosomiasis.

## Abbreviations

A/G: Albumin/globulin ratio
ALT: Alanine amino transferase
EPG: Egg number per gram of feces
M2BPGi: Mac-2 binding protein glycosylation isomer
PI: Post-infection
